# Copy number variations in patients with idiopathic recurrent pregnancy loss: an array-CGH approach

**DOI:** 10.55730/1300-0144.5511

**Published:** 2022-08-20

**Authors:** Onur YILDIZ, Fatma SILAN, Taner KARAKAYA, Öztürk ÖZDEMİR

**Affiliations:** Department of Medical Genetics, Faculty of Medicine, Çanakkale Onsekiz Mart University, Çanakkale, Turkey

**Keywords:** Array-comparative genomic hybridization, copy number variation, recurrent pregnancy loss

## Abstract

**Background/aim:**

It is not always possible to determine the causative basis of pregnancy losses and even today it has been reported that 50% of cases with recurrent pregnancy loss (RPL) have no reason to be detected. In our study, it is aimed to reveal the copy number variations (CNVs) of the genes which presumably have a potential effect in individuals with RPL and contribute to subsequent functional studies in the follow-up.

**Materials and methods:**

We retrospectively evaluated the array-comparative genomic hybridization (aCGH) data of cytogenetically 64 normal individuals (21 couples, 11 unrelated women, and 11 unrelated men) who had applied to our outpatient clinic from January 2016 to December 2017, for the history of idiopathic two or more RPL.

**Results:**

A total of 83 CNVs were detected in 56 different chromosomal regions [36% (20/56) is deletion and 64% (36/56) is duplication] in 40/64 (62.5%) of the cases. Two detected deleterious CNVs encompassing 1p36.22-p36.21 and 10q11.22 chromosomal locus have been reported as pathogenic according to the Database of Genomic Variants (DGV).

**Conclusion:**

CNVs that may play a role in the genetic etiology of idiopathic RPL were revealed in our study and potential chromosomal loci were introduced to the literature for further analysis. The detection of CNVs and their association with reproduction such as RPL, infertility, and even other diseases will allow us to have more information about the clinical consequences and will make it possible to provide more accurate and comprehensive genetic counseling.

## 1. Introduction

The 15%–25% of pregnancies end up with a miscarriage and 3%–5% of pregnancy losses are recurrent [1,2]. Recurrent pregnancy loss (RPL) is defined as the loss of two or more pregnancies before the 20th week of gestation [3–5]. However, some publications suggest that there should be three pregnancy losses for RPL and then recommend evaluation [6]. RPL is a troublesome condition for couples because the probability of miscarriage increases gradually after each pregnancy loss [7].

It is not always possible to determine the underlying cause of pregnancy losses. No causative factor could be identified in up to 50% of cases with a history of RPL [8,9]. This group is classified as idiopathic RPL. Prospective and retrospective studies reveal that women who had a previous pregnancy with loss/abortion faced an increased risk of loss in the next pregnancy [7]. RPL prevalence among the first-degree relatives of idiopathic pregnancy loss patients increased 6-fold compared to the general population [10].

Copy number variations (CNVs) are very common in the population. Some CNVs are considered benign, while some are variants that reflect the patient’s phenotype. There are also a large number of rare CNVs with unknown phenotypic consequences. As a result, the interpretation of rare variants of unknown clinical significance causes great difficulty. In this respect, international databases have been established to collect as much data as possible and to share information among experts. However, general literature information of some CNVs for the potential etiological cause associated with RPL is not sufficient. CNVs can affect the gene dosages that are critical in early gestation or disrupt segregation of chromosomes, resulting in miscarriages [11].

Large perisentromeric and subtelomeric CNVs may predispose to idiopathic RPL. In the genomes of patients with idiopathic RPL, almost twice as much CNVs [>300 kilobase (kb)] were detected compared to the controls. In particular, 63% of these large CNVs (>300 kb) in subjects with idiopathic RPL are in pericentromeric and subtelomeric regions, whereas only 33% of the large CNVs detected in control parental genomes are in perisentromeric and subtelomeric regions [12].

In order to detect the changes in the number of copies of cases with RPL, we should perform analyses at higher resolutions. CNVs can be detected using the array-comparative genomic hybridization (aCGH) technique without a need of cell culture.

With the aCGH approach, new candidate genes causing recurrent miscarriages can be found. Analysis of which pathways the detected genes belong to (thrombosis, immunological, placental development, etc.) will be useful in the follow-up of patients [12]. It will also be possible to develop new strategies on candidate genes. In this way, it will enable the patients in this group to get a more advanced and effective diagnosis.

## 2. Materials and methods

### 2.1. Patient recruitment

In this study, we evaluated the data of 64 patients (21 couples, 11 unrelated women, and 11 unrelated men) with idiopathic RPL obtained by the aCGH method in terms of their CNVs who had applied to the Çanakkale Onsekiz Mart University Medical Genetics outpatient clinic from January 2016 to December 2017. All the cases included in this study had demonstrated normal karyotypes.

All experimental procedures were conducted in accordance with the principles of the Declaration of Helsinki, and informed written consent was obtained from patients or their guardians. This was a retrospective clinical study approved by Çanakkale Onsekiz Mart University Clinical Research Ethics Committee with the decision numbered 20-06 and dated December 13, 2017.

### 2.2. DNA isolation

Genomic DNA had been extracted from the peripheral blood of all patients using QIAamp DNA Blood Mini QIAcube Kit (Qiagen, Hilden, Germany) according to the standard procedures. After the isolation, concentrations of the patient DNAs and reference DNA samples were measured with NanoPhotometer® P330 (Implen, Westlake Village, CA, USA) and then the concentrations of all samples were adjusted to 50 ng.

### 2.3. Chromosomal microarray

SurePrint G3 ISCA V2 CGH 8 × 60K (Agilent Technologies, Santa Clara, CA, USA) slide was used for the aCGH study. Labeling, prehybridization preparation, hybridization at 67 °C for 24 h, washing and slide scanning with Agilent Microarray Scan Control software (Agilent Technologies, Santa Clara, CA, USA) steps were implemented before the analysis.

### 2.4. Analysis

Feature Extraction 12.0.1.1 software (Agilent Technologies, Santa Clara, CA, USA), which also provided the quality control report of the study, was used to convert the “.tiff” files to mathematical data after scanning. Agilent CytoGenomics 4.0 software (Agilent Technologies, Santa Clara, CA, USA) was utilized for the analysis.

The mean log ratios of at least three probes were determined as a threshold limit > 0.5 for a duplication and <–0.5 for a deletion.

### 2.5. Statistics

The data were analyzed using IBM SPSS Statistics for Windows, version 20.0 (IBM Corp, Armonk, NY, USA). Percentage, median, mean, standard deviation (SD), minimum, and maximum were used for the identification of descriptive data. The detected CNVs were compared with the Database of Genomic Variants (DGV). Chi-squared and ANOVA tests were used for statistical comparison of the groups. In cases where the assumptions of the ANOVA test were not met, the Kruskal–Wallis nonparametric test was done. As a result of statistical comparison of CNVs, regions with p < 0.05 were considered statistically significant.

## 3. Results

The characteristics of our cases with idiopathic RPL are presented in Table 1.

Mean age and median of the study group was 33.9 ± 6.6 and 33.5 (min: 22.0; max: 55.0) years respectively. The number of female and male patients included in the study is both 32; the mean age is 32 years for the female, 36 years for the male.

**Figure 1:**
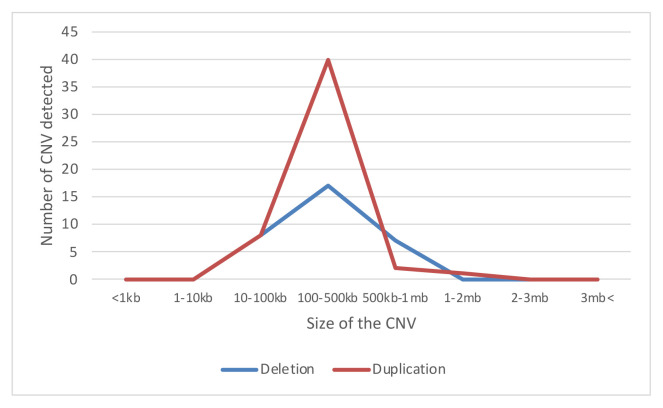
CNV distributions according to the sizes and mutation types (deletion or duplication) detected in our individuals with idiopathic RPL.

**Figure 2:**
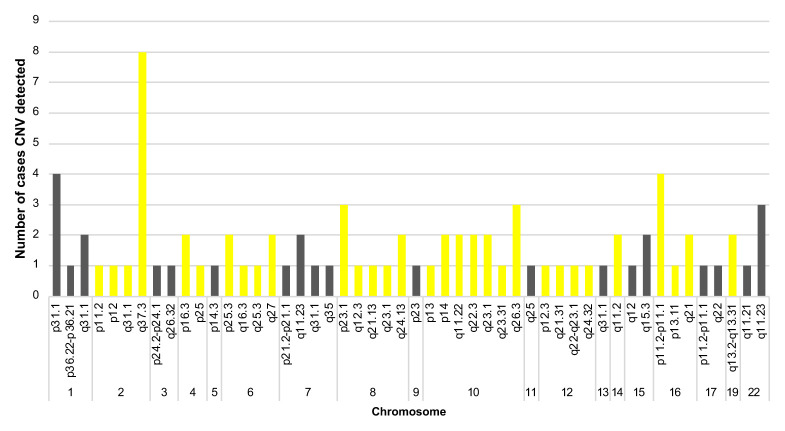
Distribution of CNVs according to the chromosomal loci detected in our individuals with idiopathic RPL.

**Figure 3:**
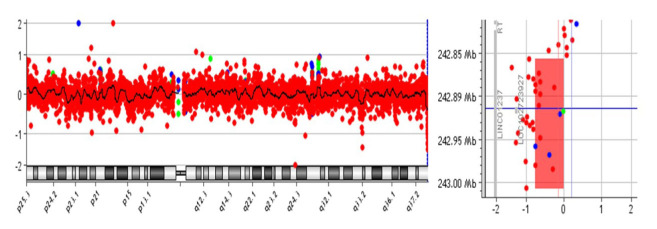
aCGH image of heterozygous deletion (150Kb) containing *LINC01237, LOC102723927* genes at 2q37.3 locus in eight cases with a history of RPL.

**Figure 4:**
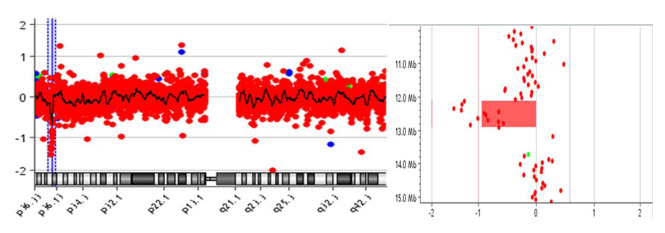
aCGH image of p36.22-p36.21 locus at chromosome 1 containing *TNFRSF8, TNFRSF1B, DHRS3, MIR7846, MIR4632, VPS13D, SNORA59A, SNORA59B, MIR6730, AADACL4, AADACL3, C1orf158, PRAMEF12, PRAMEF1, PRAMEF11, HNRNPCL1, HNRNPCL3, HNRNPCL4* in a case with RPL history shows heterozygous deletion (792 Kb).

**Figure 5:**
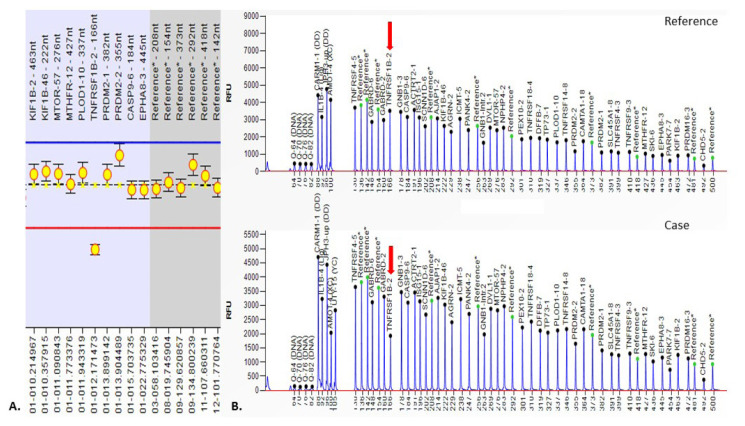
MLPA analysis showing the heterozygous deletion of *TNFRSF1B* gene located in 1p36.22-p 36.21 (A and B).

Significant CNVs were detected in 40 (~62%) of total 64 individuals (Table 2). In these 40 cases, a total of 83 CNVs were detected in 56 different regions [36% (20/56) is deletion and 64% (36/56) is duplication (Figure 1)]. There were no CNVs detected in the 18, 20, 21, X, and Y chromosomes (Figure 2). It was observed that the detected CNVs were generally spanning between 100 and 500 kb, and no CNV was detected below 10 kb and above 2 megabase (Mb). CNV containing *LINC01237* and *LOC102723927* gene regions was detected in eight individuals as it was the most common CNV in our patient group (Figure 3). In one case, a heterozygous deletion was detected in the 10q11.22 chromosome region containing *GPRIN2*, *NPY4R*, *ANXA8* genes, and in another case, a heterozygous deletion was observed including *TNFRSF8*, *TNFRSF1B*, and *DHRS3* genes in the 1p36.22-p36.21 chromosome region (Figure 4) further confirmed with SALSA MLPA P147 1p36 probemix (MRC-Holland BV, Amsterdam, the Netherlands) (Figure 5). These two CNVs have been reported as pathogenic in many databases (ClinVar and DGV etc.). Other remaining detected CNVs have been regarded as benign and possible benign in the literature.

Genes detected in CNV regions in two or more cases were evaluated by comparing with the genes detected in the population ratios in DGV database (Table 3). These genes include *LINC01237*, *LOC102723927*, *ZRANB2-AS*, *LINC01566*, *NEGR1*, *FRG2DP*, *CYP2E1*, *DUSP22*, *CATSPER2*, *UPK3B*, *POMZP3*, *PSG8*, *PSG1*, *PSG6*, *PSG7*, *PSG11*, *PSG2*, *PSG5*, *PSG4*, and the CNVs including these gene regions have been evaluated as benign or probable benign in ClinVar.

**Figure 6:**
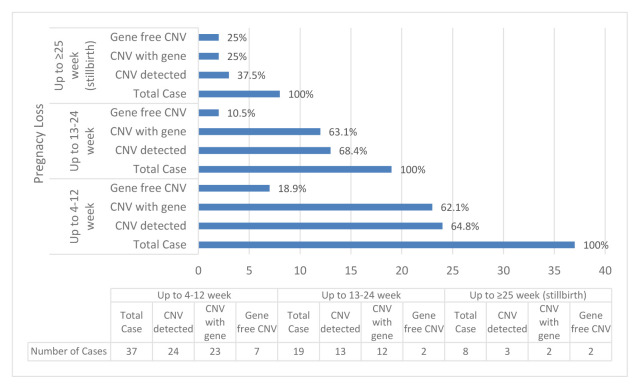
CNV distributions according to the week of pregnancy loss detected in our individuals with idiopathic RPL.

In cases with up to 4–12-week pregnancy loss, the number of cases with CNV was 24 (64%), and 23 (62%) of these cases with CNV regions contained genes, 7 (18%) cases had gene-free CNV regions. Six of these cases had both gene-containing region and gene-free region. In cases with up to 13–24-week pregnancy loss, the number of cases with CNV was 13 (68%), and 12 (63%) of these cases with CNV regions contained genes, 2 (10%) cases have gene-free CNV regions. A single case had both gene-containing region and gene-free region in this group. In cases with up to ≥25-week pregnancy loss (stillbirth), the number of cases with CNV was 3 (37%) and 2 (25%) of these cases with CNV regions contained genes, 2 (25%) cases had gene-free CNV regions. A single case had both gene-containing region and gene-free region in this group (Figure 6).

**Figure 7:**
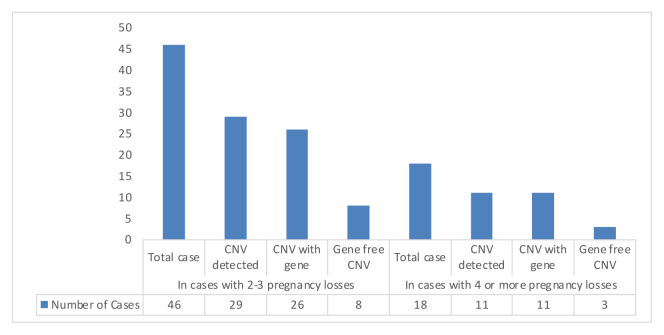
CNV distributions according to the number of pregnancy loss detected in our individuals with RPL.

In cases with 2–3 pregnancy losses, the number of cases with CNV was 29 (63%) and 26 (56%) of these cases with CNV regions contained genes, 8 (17%) cases had gene-free CNV regions. Five of these cases had both gene-containing region and gene-free region. In cases with 4 or more pregnancy losses, the number of cases with CNV was 11 (61%) and 11 (61%) of these cases with CNV regions contained genes, 3 (16%) cases had gene-free CNV regions. Three of these cases had both gene-containing region and gene-free region. There were no significant differences between the cases with 2–3 pregnancy losses and those with 4 or more pregnancy losses in terms of detected CNV number (Figure 7).

## 4. Discussion

The 1%–3% of couples wanting to have a child may encounter RPL. RPL is a polygenic, multifactorial health burden with anatomical, endocrine, immunological, infectious, thrombophilic (both acquired and congenital), genetic, and environmental basis in the etiology [13]. The main first-tier genetic tests performed to elucidate causative factors of RPL are chromosome analysis of the peripheral blood sample of women and men, and SNP analysis of *FV* and *F2* genes of women in terms of thrombophilia [14]. Even if all of these factors are examined, approximately 50% of underlying causes of RPL remains idiopathic. Therefore, further comprehensive studies are needed to determine the etiology of RPL.

aCGH, or so-called chromosomal karyotyping, is a high-resolution and genome-wide analysis method enabling to detect CNVs within DNA sequence. Most CNVs are polymorphic. The genome content of any two individual may vary up to 50–100 Mb due to CNV polymorphism. CNVs are important because they lead to a couple of consequences in chromosomal rearrangements and they can also affect the phenotype depending on whether they contain a gene or not. To date, such a few studies have been conducted to evaluate CNVs in the cases with RPL [12,15–17].

In the study by Rajcan-Separovic et al., 22 individuals were studied with the aCGH method (Agilent® 105K microarray). Eleven previously undetected CNVs were found on 8 pairs, 13 fetal materials. In our study, the CNV regions were not detected in the same chromosomal regions compared with the CNVs detected in that study; this may be due to the limited number of cases included for the study or the ethnic difference between two patient groups [15].

A total of 558 cases of RPL and 205 healthy female controls were included in the study by Nagirnaja et al., albeit no male control subjects were recruited. In their study, a new duplication was detected in the 5p13.3 chromosome region and it was suggested that changes in the expression of *PDZD2* and *GOLPH3* genes may be a risk factor increasing pregnancy complications [16]. In our study, no variations were detected in these regions. The study of Nagirnaja et al. has the largest patient and control group in the medical literature, and our sample group is relatively small.

Twenty-five cases with idiopathic RPL were included in the study conducted by Kasak et al., and maternal, paternal, and placental genetic factors were investigated. *NUP98* and *MTRR* genes have been shown to be effective in cases with RPL, and it has been reported that large pericentromeric and subtelomeric CNVs may be risk factors for RPL. Although no variation in these genes was detected in our study, CNVs in pericentromeric and subtelomeric regions were also detected in our study [12].

The 16 couples and 12 female patients with RPL were included and analyzed by cytogenetic and microarray tests in the study conducted by Karim et al. in 2017. Microdeletion and/or microduplication were detected in the 8p23.1, 10q11.21-q11.22 and 15q11.2 chromosomal regions in at least 10% of the patients included in the study and the dosage of *GSTT1*, *CTLAPL*, *HLA*, and *MSR1* genes residing 22q11.23, 3p22.2, 6p21.32, and 8p22 chromosomal locations respectively have been reported to be affected [17]. In our study, CNVs were also detected in the chromosomal regions of 8p23.1, 10q11.21-q11.22 and it should be remembered that these regions may pose a risk for RPL.

The number of our cases having dosage difference in *LINC01237*, *LOC102723927*, *ZRANB2-AS*, *LINC01566*, *NEGR1*, *FRG2DP*, *CYP2E1*, *DUSP22*, and *CATSPER2* genes were found to differ statistically significantly compared to DGV database (p < 0.05). *UPK3B*, *POMZP3*, *PSG8*, *PSG1*, *PSG6*, *PSG7*, *PSG11*, *PSG2*, *PSG5*, and *PSG4* genes have not been statistically significantly different according to the DGV (p > 0.05). It is predicted that this may be due to the selection of cases in the DGV database, and it is necessary to conduct studies with healthy control groups known to be fertile to evaluate CNVs containing these genes.

*LINC01237* gene is extensively expressed in appendix, lymph node, spleen, thyroid and endometrium tissues [18]. Although the clinical significance of deletion in the *LOC102723927* gene region has not been fully established, it has been suggested that it may be associated with uncontrolled gestational diabetes [19]. These genes are long noncoding RNA genes, and the fact that the *LINC01237* gene is expressed in appendix, lymph node, and spleen suggests that it may play a role in the immune system. The fact that it is also extensively expressed in the endometrium supports that it can be a particularly important candidate gene in the etiology of pregnancy losses.

Expression of the *SYCE1* gene product is most intensely from the testicle and limitedly from the placenta, brain, and prostate, respectively [20]. CNV in this region is classified as VUS (variant of unknown significance) in ClinVar and was detected in patients with a history of preeclampsia and normal delivery [19]. Vries et al. in 2014 detected a nonsense homozygous mutation (Q205X) at the *SYCE1* gene in two sisters with premature ovarian failure [21]. In 2015, Maor-Sagie et al. revealed a homozygous mutation at the *SYCE1* gene in two Iranian brothers and associated it with a spermatogenesis error [22]. In a study of 970 Chinese men with nonobstructive azoospermia, Huang et al. determined 134 kb deletion at the *SYCE1* gene in three individuals and reported that it had been associated with nonobstructive azoospermia [23].

Avidan et al. detected 70 kb deletion in *CATSPER2* gene in three brothers and reported that this was related to nonsyndromic male infertility [24]. In a study reported in 2013, Hoppman et al. found the percentage of those carrying the heterozygous deletion of this gene with a frequency of 1%; in one patient, they detected the homozygous deletion of this gene and concluded that the deletions of this region were associated with hearing loss and male infertility [25]. Jaiswal et al. reported the deletion of this gene in two infertile brothers as a case report [26].

The CNV containing *PSG8*, *PSG1*, *PSG6*, *PSG7*, *PSG11*, *PSG2*, *PSG5*, *PSG4* have been evaluated as likely benign/benign in ClinVar database. Genes in this region are expressed in the placenta. In a study conducted by Arnold et al. in 1999, there was a relationship between *PSG11* gene and the risk of recurrent pregnancy loss [27].

*FER1L6-AS2* is largely expressed in the stomach, while as a 2nd place it is expressed from the testicle. In the study published by Ledig et al. in 2010, the *FER1L6* gene was reported to be associated with folliculogenesis and male infertility [28].

In our study, sex of patients and gene-organ expressivities were evaluated and *CATSPER2*, *LINCO1208*, *FAM27E5*, *FLJ36000*, *IMMP2L*, *PIWIL3*, *SYCE1*, *FRG2DP*, *TP53TG3HP*, *ZRNAB2-AS2*, and *LINCO1566* were evaluated as possible paternal factors and *UPK3B*, *POMZP3*, *PSG8*, *PSG1*, *PSG6*, *PSG7*, *PSG11*, *PSG2*, *PSG5*, *PSG4*, *ZNF595*, *ZNF718*, *ZFPM2*, *ZFPM2-AS1*, *ZDHHC14*, *HAGLR*, *HAGLROS*, *LINC01237*, *LOC102723927*, *DUSP22*, and *GGT3P* as possible maternal factors.

Our study has a limitation. Due to the fact that we analyzed retrospectively the data of individuals with idiopathic RPL, it is not always possible to declare that detected CNVs are de novo or inherited because of the lack of CNV profiles of the placental tissues. To get more valid information synchronous (mother-father-placenta) CNV profile analyses of RPL cases are needed.

## 5. Conclusion

There has not been enough study for the CNVs detected by aCGH approach in the possible genetic basis of RPL. Genes that may play a role in the genetic etiology of RPL were revealed in our study and new candidate genes were introduced to the literature for further analysis. In addition, genetic factors belonging not only to the mother but also to the father were found to be important in the research of genetic basis. Expanding and increasing research on RPL in our country will contribute to the elucidation of genetic causative factors. At the same time, the detection of CNVs and their association with reproduction such as RPL, infertility, and even other diseases will make us have more information about the clinical consequences and will make it possible to provide more accurate and comprehensive genetic counseling.

## Figures and Tables

**Table 1:** Demographic characteristics of our cases with idiopathic RPL.

	n (%)
**Sex**	
Female	32 (50.0)
Male	32 (50.0)
**Level of education**	
Illiterate/No formal education	1 (1.7)
Up to primary school	10 (16.9)
Up to middle school	8 (13.6)
Up to high school	19 (32.2)
University	21 (35.6)
**Individual having children alive**	
Yes	27 (42.9)
No	36 (57.1)
**Family history of RPL**	
Present	8 (12.5)
Absent	56 (87.5)
**History of congenital anomaly in the family**	
Present	12 (18.8)
Absent	52 (81.3)
**Family history of learning disabilities**	
Present	6 (9.4)
Absent	58 (90.6)
**Consanguineous marriages**	
Yes	12 (18.8)
No	52 (81.3)

**Table 2:** CNV distributions of our subjects with idiopathic RPL.

	CNV not detected n (%)	CNV detected n (%)	CNV with gene n (%)	Gene-free CNV n (%)	CNV both with gene and gene-free n (%)
Number of cases	24 (37.5)	40 (62.5)	37 (57.8)	3 (4.6)	11 (17.1)
Number of male cases	10 (15.6)	22 (34.3)	20 (31.2)	2 (3.1)	5 (7.8)
Number of female cases	14 (21.8)	18 (28.1)	17 (26.5)	1 (1.5)	6 (9.3)

**Table 3:** Comparing the genes detected in our study with the ratios in DGV database.

GENES		Our study (number of detected cases/total case)	DGV database (number of detected cases/total case)	p	Reference
*LINC01237, LOC102723927*	Del	8/64	41/771	0.020	dgv165e55
*ZRANB2-AS2*	Dup	4/64	1/112	0.003	nsv818211
*LINC01566*	Dup	4/64	13/29084	0.0001	dgv2963n100
*NEGR1*	Dup	3/64	2/1557	0.0001	dgv19n27
*FRG2DP*	Dup	3/64	189/29084	0.009	dgv2965n100
*CYP2E1*	Dup	3/64	110/29084	0.002	dgv1001n100
*DUSP22*	Del	2/64	84/29084	0.015	dgv5878n100
*UPK3B, POMZP3*	Del	2/64	3/112	0.514	dgv63n64
*CATSPER2*	Del	2/64	17/29084	0.002	dgv2585n100
*PSG1, PSG2, PSG4, PSG5, PSG6, PSG7, PSG8, PSG11*	Dup	2/64	16/771	0.384	dgv140e55

p: Binomial test; del: deletion; dup: duplication

Values in bold are admitted statistically significant (p < 0.05).
